# Visualization of the Evolution and Transmission of Circulating Vaccine-Derived Poliovirus (cVDPV) Outbreaks in the African Region

**DOI:** 10.21769/BioProtoc.5376

**Published:** 2025-07-05

**Authors:** Collins D. Owuor, Brook Tesfaye, Arthur Yannick Doungmo Wakem, Sakma Kabore, Caroline Obianuju Ikeonu, Mariette Egbonoumi Fifatin Glitho Epse Doussoh, Priscilla Epse Mosoke Bobimwoh Sigala, Idris Ibrahim Ibrahim, Abdullateef Jimoh, Idah Ndumba, Jermaine Khumalo, David O. Oviaesu, Cheroigin Kipchirchir, Carolyne Gathenji, John Kipterer, Kebba Touray, Hamisu Abdullahi, Kathleen Rankin, Ousmane M. Diop, Julius E. Chia, Ndoutabe Modjirom, Jamal A. Ahmed, Anfumbom K.W. Kfutwah

**Affiliations:** 1 Polio Eradication Program, Office of the Regional Director, World Health Organization, Regional Office for Africa, Brazzaville, Republic of the Congo; 2 Gates Foundation, Seattle, WA, USA; 3 Polio Eradication Program, World Health Organization, Geneva, Switzerland

**Keywords:** Poliovirus, cVDPV, phylogeography, VP1 gene, African Region (AFRO)

## Abstract

Since the creation of the Global Polio Eradication Initiative (GPEI) in 1988, significant progress has been made toward attaining a poliovirus-free world. This has resulted in the eradication of wild poliovirus (WPV) serotypes two (WPV2) and three (WPV3) and limited transmission of serotype one (WPV1) in Pakistan and Afghanistan. However, the increased emergence of circulating vaccine-derived poliovirus (cVDPV) and the continued circulation of WPV1, although limited to two countries, pose a continuous threat of international spread of poliovirus. These challenges highlight the need to further strengthen surveillance and outbreak responses, particularly in the African Region (AFRO). Phylogeographic visualization tools may provide insights into changes in poliovirus epidemiology, which can in turn guide the implementation of more strategic and effective supplementary immunization activities and improved outbreak response and surveillance. We created a comprehensive protocol for the phylogeographic analysis of polioviruses using Nextstrain, a powerful open-source tool for real-time interactive visualization of virus sequencing data. It is expected that this protocol will support poliovirus elimination strategies in AFRO and contribute significantly to global eradication strategies. These tools have been utilized for other pathogens of public health importance, for example, SARS-CoV-2, human influenza, Ebola, and Mpox, among others, through real-time tracking of pathogen evolution (https://nextstrain.org), harnessing the scientific and public health potential of pathogen genome data.

Key features

• Employs Nextstrain (https://nextstrain.org), which is an open-source tool for real-time interactive visualization of genome sequencing datasets.

• First comprehensive protocol for the phylogeographic analysis of poliovirus sequences collected from countries in the World Health Organization (WHO) African Region (AFRO).

• Phylogeographic visualization may provide insights into changes in poliovirus epidemiology, which can in turn guide the implementation of more strategic and effective vaccination campaigns.

• This protocol can be deployed locally on a personal computer or on a Microsoft Azure cloud server for high throughput.

## Background

Significant progress has been made toward a poliovirus-free world since the creation of the Global Polio Eradication Initiative (GPEI) in 1988 [1]. GPEI efforts have resulted in the eradication of wild poliovirus (WPV) serotypes two (WPV2) and three (WPV3) and limited transmission of serotype one (WPV1) in Pakistan and Afghanistan [1]. However, the increased emergence of circulating vaccine-derived poliovirus (cVDPV) outbreaks and the continued circulation of WPV1 pose a continuous threat of international spread [2,3]. For example, WPV1 viruses showing a direct link to the viruses circulating in Pakistan have been isolated from stool samples collected in Malawi and Mozambique in November 2021 and March 2022, respectively ( https://www.who.int/emergencies/disease-outbreak-news/item/wild-poliovirus-type-1-(WPV1)-malawi ). Additionally, cVDPV serotype 2 (cVDPV2) isolates from environmental samples in five countries in the WHO European Region (EURO) have recently been genetically linked to cVDPV2 strains circulating in the World Health Organization (WHO) African Region (AFRO) [4].

The importation of WPV1 into WHO AFRO and the exportation of cVDPV2 from WHO AFRO to WHO EURO highlight the need to further strengthen surveillance and outbreak responses. Furthermore, there is a need for novel strategies to guide the implementation of more strategic and effective vaccination campaigns. Genetic sequencing of cVDPVs from stool and wastewater samples is a useful tool employed for the confirmation of poliovirus detection, identification of cVDPV origins, and tracking of geographic spreading patterns [5,6]. It is also important for the determination of appropriate and most effective vaccination strategies [6]. For example, it is possible to infer which cVDPV outbreak cases are due to local transmissions and which are more likely to be linked to chains of cVDPV transmission in other countries. Phylogeographic methods are based on Nextstrain real-time tracking of pathogen evolution (https://docs.nextstrain.org/en/latest/index.html) [7] and Bayesian evolutionary analysis sampling trees (BEAST) (https://beast.community/about) to infer the origins and geographic spread of cVDPVs between and within WHO AFRO countries [5]. These methods employ discrete trait analysis (DTA), which can provide information on the geographic history of viral spread and associated rates of transmission [5]. Starting from geolocated virus sequences, this approach allows inference of the location of internal nodes in phylogenetic trees [5,7]. DTA has previously been applied to inform viral diffusion for several infectious diseases, including SARS-CoV-2, human and animal influenza, Mpox, and Ebola (https://nextstrain.org), and is considered a powerful tool for molecular epidemiology of pathogens.

Regular updates of the cVDPV surveillance sequence data using phylogeographic visualization tools may provide insights into changes in poliovirus epidemiology, which can in turn guide the implementation of more strategic and effective supplementary immunization activities and improved outbreak response and surveillance. These analyses may be particularly relevant to halt the ongoing outbreaks of cVDPVs in WHO AFRO through improved surveillance and timely and effective outbreak response, possibly achieving the global poliovirus eradication efforts of the GPEI. With this goal in mind, we created a comprehensive protocol for the phylogenetic and phylogeographic analysis of poliovirus sequence datasets using Nextstrain [7], a powerful open-source tool for the real-time interactive visualization of genome sequencing data. Approaches for phylogeographic visualization of poliovirus in WHO AFRO are detailed in this protocol.

## Equipment

We used shell commands on a Linux-based operating system with superuser privileges.

**Computational requirements**: We used a workstation or a server with a 64-bit Linux-based operating system, possessing 12 GB RAM and sufficient hard disk space (at least 500 GB) to store the files used and produced in the analysis. The commands given in this analysis protocol were validated on Ubuntu (24.04 LTS) Linux Distribution running on a Microsoft Azure cloud server (https://azure.microsoft.com/en-gb) and on a personal laptop running the same system.

## Software and datasets

1. Required software

This protocol uses the following tools and Nextstrain software to perform the phylogeographic analysis:

a. Anaconda ( https://github.com/conda/conda )

b. Nextstrain https://github.com/nextstrain [7]

c. Augur https://github.com/nextstrain/augur [7]

d. Auspice https://github.com/nextstrain/augur [7]

e. MAFFT (https://github.com/GSLBiotech/mafft [8]

f. IQTREE http://www.iqtree.org [9]

All requisite tools and their dependents were installed (personal computer or workstation) before proceeding with the analysis.

2. Datasets

The protocol uses the poliovirus VP1 gene sequence datasets made publicly available by the National Center for Biotechnology Information (NCBI): https://www.ncbi.nlm.nih.gov/nucleotide/ for development. Similar datasets are routinely generated in AFRO-supported laboratories in WHO AFRO under the supervision of the global network of poliovirus laboratories (Global Poliovirus Laboratory Network, GPLN).

The installation steps for all tools used in this protocol and the instructions for downloading the requisite datasets are given in the following section.

## Procedure

**Figure 1.**
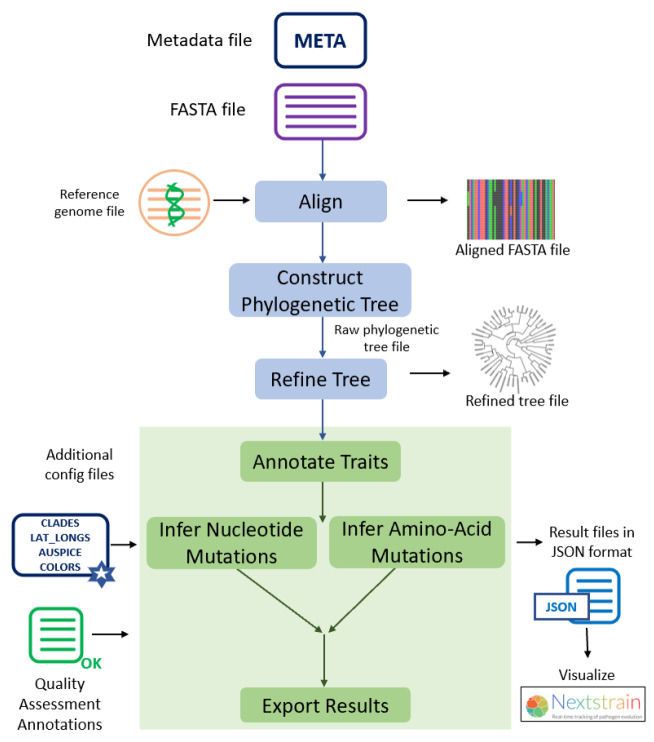
Different steps described in this protocol and the Augur modules used in each of the analysis steps

The individual steps involved in this protocol and the Augur modules used in each step are summarized in **Figure 1**.

**A. Install Anaconda**

Anaconda is an open-source distribution of Python that simplifies the management of Python packages and environments. To install Anaconda, use the following commands:

$ wget https://repo.anaconda.com/miniconda/Miniconda3-latest-Linux-x86_64.sh

$ echo 'exportPATH="~/miniconda3/bin:$PATH"' >> ~/.bashrc

Follow the on-screen instructions to continue the installation. The **anaconda3** folder can be found in the directory shown in the installer script (Miniconda3-latest-Linux-x86_64.sh). The installation is then activated and tested by running the following commands:

$ source ~/.bashrc

$ conda list

**B. Install Nextstrain command-line interface (Nextstrain-CLI)**

Use the commands below to install Nextstrain directly in a Conda environment.

$ conda create -n nextstrain

$ conda activate nextstrain

$ curl -fsSL --proto '=https' https://nextstrain.org/cli/installer/linux | bash

$ nextstrain setup --set-default conda

Enter the interactive Nextstrain shell in the current directory (.).

$ nextstrain shell .

To check whether Nextstrain was successfully installed, use the following command:

$ nextstrain version

The version number shown in the output should be 8.5.3 or higher. Augur is a bioinformatics toolkit for phylogenetic analysis, whereas Auspice is an interactive visualization tool for phylogenomic data [7].

Test Augur and associated Auspice for visualization:

$ augur --help

$ auspice --help

**C. Test MAFFT and IQ-TREE installations**

MAFFT (multiple alignment using fast Fourier transform) is required by Augur to perform multiple-sequence alignments [8]. IQ-TREE is an open-source tool for constructing maximum-likelihood trees using phylogenetic data [9]. IQ-TREE is required by Augur for constructing a phylogenetic tree from sequence data. To test these two installations, use the following command:

$ mafft - - help

$ iqtree - - help

**D. Download poliovirus Sabin 2 vaccine reference**

Before proceeding with the analysis, download the Sabin 2 vaccine reference strain from NCBI in GenBank format (https://www.ncbi.nlm.nih.gov/genbank/). For this analysis, the strain with the accession number AY082679 is downloaded.

**E. Download publicly available VP1 gene sequence dataset**

Sequence datasets are downloaded from NCBI (https://www.ncbi.nlm.nih.gov/nucleotide/) in FASTA format using the accession numbers provided in **File S1**.

**F. Preparation of input files**

To use Nextstrain for phylogenetic analysis and visualization, prepare the following input files (**Table 1**):

**Table 1.** List of input files required to run the phylogeographic analysis pipeline.

File	Description
**Required input files**	
polio_sequences.fasta	Collection of VP1 gene sequences to be analyzed in FASTA format
polio_metadata.tsv	Tab-delimited text file describing all sequences in the polio_sequences.fasta file
AY082679.gb	Poliovirus Sabin 2 reference genome in GenBank format
**Additional configuration files**	
auspice_config.json	Text file in JSON format specifying visualization settings
lat_longs.tsv	Tab-delimited text file for displaying geographic traits
colors.tsv	Tab-delimited file containing hex color codes for metadata elements

1. polio_sequences.fasta

**Figure 2.**
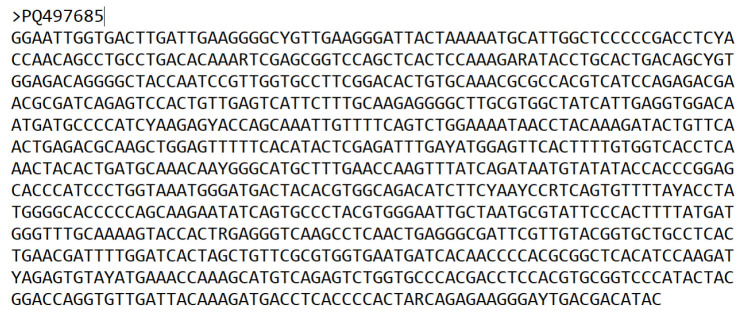
Sample record for poliovirus accession number PQ497685 strain in the sequences.fasta format

This is the single FASTA file containing a collection of poliovirus sequences to be analyzed. For this analysis, use the sequence dataset downloaded from NCBI. Each sequence in the FASTA file has the unique strain identity of the virus as the sequence header. A sample sequence record for the FASTA file is shown in **Figure 2**.

2. polio_metadata.tsv

A tab-delimited metadata file that describes the sequences given in the FASTA file. The various fields included in the metadata file are as follows:

a. Required fields: strain, virus, date.

For each strain ID in the polio_sequences.fasta file, there should be an entry under the strain column in the metadata file.

b. Additional fields (if using published data): Accession, Authors, URL, Title, Journal, Paper_URL.

c. To infer ancestral traits, additional information fields such as continent, region, country, province, state, and city should be included in the metadata file if available.

3. auspice_config.json

This file is needed to set various display options for visualization. A sample config file is available as **File S2**.

4. lat_longs.tsv

**Figure 3.**
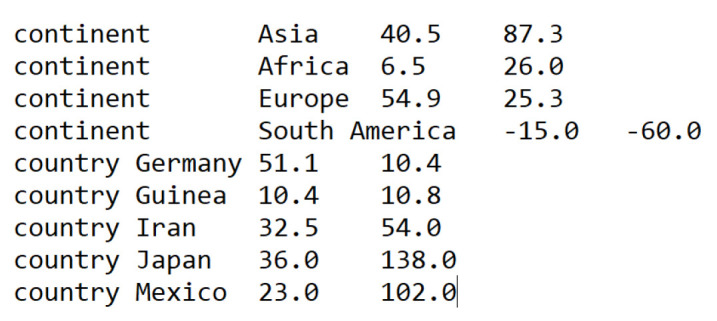
Summary screenshot of the lat_longs.tsv file required by Nextstrain for visualizing geographic traits

A tab-separated file containing latitudes and longitudes for all regions, countries, states, and cities in the dataset (**Figure 3**). This file is used to display geographic traits during visualization.

## Data analysis

**A. Alignment to the reference genome**

Augur uses MAFFT to perform multiple-sequence alignments. To create an alignment file using Augur, use the following command:

$ augur align --sequences <polio_sequences.fasta> --reference-sequence <AY082679.gb> --output <aligned_polio.fasta> --fill-gaps –remove reference --nthreads <8>

**B. Constructing the phylogenetic tree**

Augur uses IQTREE as the default software to construct a phylogenetic tree from the multiple-sequence alignment file. The branch lengths in the tree are a measure of nucleotide divergence.

The following command will generate a phylogenetic tree in Newick format (.nwk):

$ augur tree --alignment <aligned_polio.fasta> --output <raw_tree_polio.nwk> --nthreads <8>

**C. Refining the phylogenetic tree**

The raw tree constructed in the previous step is further processed by Augur using TreeTime to adjust the branch lengths according to the sampling dates of the sequences. In the analysis, the root of the tree is specified by giving the sequence name AY082679.1 explicitly with the --root parameter of the refine command. The --clock-rate parameter is used to run the analysis using a fixed evolutionary rate to produce a robust time-resolved phylogeny, and the --clock-filter-iqd parameter filters out genes that do not follow the evolutionary rate or molecular clock. For poliovirus genomes, this rate is fixed at 0.01 or 1 × 10^-2^ substitutions per site per year. To produce a time-resolved tree, use the following command:

$ augur refine --tree <raw_tree_polio.nwk> --alignment <aligned_polio.fasta> --metadata <polio_metadata.tsv> --output-tree <refined_polio_tree.nwk> --output-node-data <branch_lengths_polio.json> --timetree --coalescent opt --clock-rate 0.01 --clock-std-dev 0.04 --date-inference marginal --divergence-unit mutations --date-confidence --clock-filter-iqd 4 –-stochastic-resolve

**D. Annotating ancestral traits**

Augur can use the time tree to infer the region and country of all internal nodes. The ancestral traits for all nodes can be annotated using the following command:

$ augur traits --tree <refined_polio_tree.nwk> --metadata <polio_metadata.tsv> --output <polio_traits.json> --columns continent country --confidence --sampling-bias-correction 2.5

**E. Inferring ancestral sequences and nucleotide mutations**

The following command identifies the nucleotide mutations of the branches of the tree and infers the ancestral strain of each node:

$ augur ancestral --tree <refined_polio_tree.nwk> --alignment <aligned_polio.fasta> --output-node-data <polio_nt_muts.json> --inference joint --infer-ambiguous

**F. Inferring amino acid mutations**

The following command identifies the amino acid mutations using the reference genome and ancestral sequences:

$ augur translate --tree <refined_polio_tree.nwk> --ancestral-sequences <polio_nt_muts.json> --reference-sequence < AY082679.gb> --output <polio_aa_muts.json>

**G. Exporting output files for visualization**

The following command exports all output files generated in the previous steps of the analysis as a single JSON file to visualize the data using Nextstrain:

$ augur export v2 --tree <refined_polio_tree.nwk> --metadata <polio_metadata.tsv> --node-data <branch_lengths_polio.json> <polio_aa_muts.json> <polio_nt_muts.json> <polio_traits.json> --auspice-config auspice_config.json --lat-longs lat_longs.tsv --colors colors.tsv --output auspice/polio_global.json

**H. Viewing the data**

To visualize the output, use the following command:

$ nextstrain view auspice/ --allow-remote-access

**Figure 4.**
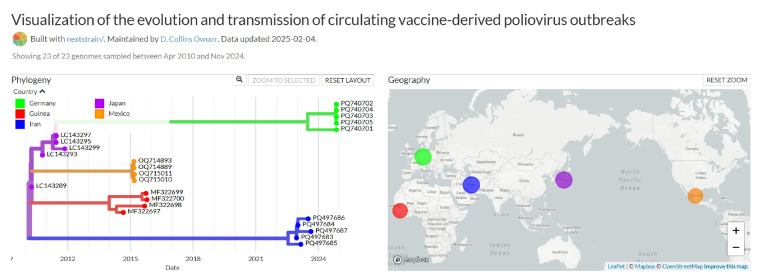
Nextstrain visualization output showing phylogeny and inferred transmissions

This command starts the Auspice server on port 4000. The output is visualized through a browser by navigating to http://127.0.0.1:4000/ or using the IP address of the machine on which the Auspice service is running and navigating to http://IP_ADDRESS_OF_MACHINE:4000/ to display the interactive Auspice page (**Figure 4**). For the links, follow the steps given at https://docs.nextstrain.org/en/latest/learn/augur-to-auspice.html. The hyperlinks correspond to a locally operated server through Auspice (installation and instructions are detailed in the protocol), which helps the user to view the phylogeny on their own system through a browser.

For the example of the visualization of the evolution and transmission of cVDPV outbreaks provided in this protocol, a visual representation of five continents is provided, color-coded by country of sequence collection. The phylogenetic tree (Phylogeny) is shown alongside the geographical view (Geography). The time-resolved phylogenetic tree shows the inferred dates on the X-axis and inferred poliovirus evolution on the Y-axis. This inferred phylogeographic representation may provide insights into changes in poliovirus epidemiology, which can in turn guide the implementation of more strategic and effective supplementary immunization activities and improved outbreak response and surveillance when focused on specific geographical locations of interest at regional, national, or sub-national levels of investigation, for example, the inference of regional patterns of poliovirus transmission within a continent. Additionally, it may provide improved surveillance, timely and effective outbreak response when combined with other epidemiological tools (e.g., vaccination coverage) and climatic and population movement datasets.

## Validation of protocol

This protocol or parts of it has been used and validated in the following research article(s):

• Hadfield et al. [7]. Nextstrain: real-time tracking of pathogen evolution. *Bioinformatics*. 34(23): 4121–4123. https://doi.org/10.1093/bioinformatics/bty407 (Figure 1).

## General notes and troubleshooting

The protocol is based on a validated pipeline developed by Nextstrain https://nextstrain.org/. The Data analysis section provides sufficient information regarding validation.

Possible software, installation, and command-line issues may arise due to software and hardware incompatibility and software versions. Additional issues may include input file configuration, which should follow the format described in the protocol. Possible troubleshooting questions and answers are addressed in the Nextstrain documentation available at https://docs.nextstrain.org/en/latest/reference/faq.html.

## Supplementary information

The following supporting information can be downloaded here:

1. File S1. Poliovirus tab-delimited text file describing all sequences in the “polio_sequences.fasta” file (polio_metadata.tsv).

2. File S2. Text file in JSON format specifying visualization settings (auspice_config.json).

## References

[1] World Health Organization(WHO). The Global Polio Eradication Initiative. Geneva: WHO.[Accessed: 29 Jan 2025]. Available from: https://polioeradication.org/.

[2] World Health Organization(2020). Circulating vaccine-derived poliovirus– Global Polio Eradication Initiative. Geneva: World Health Organization.

[3] Global Polio Eradication Initiative(2024). cVDPV2 outbreaks and the type 2 novel oral polio vaccine(nOPV2). Geneva, Switzerland: World Health Organization. https://polioeradication.org/wp-content/uploads/2024/08/GPEI_nOPV2_Factsheet_5-January-2024.pdf

[4] Böttcher S., Kreibich J., Wilton T., Saliba V., Blomqvist S., Al-Hello H., Savolainen-Kopra C., Wieczorek M., Gad B., Krzysztoszek A., .(2025). Detection of circulating vaccine-derived poliovirus type 2(cVDPV2) in wastewater samples: a wake-up call, Finland, Germany, Poland, Spain, the United Kingdom, 2024. Eurosurveillance. 30(3): e2500037. 10.2807/1560-7917.es .2025.30.3.2500037 PMC11914958 39850005

[5] Jorgensen D., Pons-Salort M., Shaw A. G. and Grassly N. C. (2020). The role of genetic sequencing and analysis in the polio eradication programme. Virus Evol. 6(2): e1093/ve/veaa040. 10.1093/ve/veaa040 PMC7409915 32782825

[6] World Health Organization(WHO). Global Polio Eradication Initiative. Reporting and classification of vaccine-derived polioviruses. https://polioeradication.org/about-polio/the-virus/vaccine-derived-polioviruses/

[7] Hadfield J., Megill C., Bell S. M., Huddleston J., Potter B., Callender C., Sagulenko P., Bedford T. and Neher R. A. (2018). Nextstrain: real-time tracking of pathogen evolution. Bioinformatics. 34(23): 4121 4123 4123. 10.1093/bioinformatics/bty407 29790939 PMC6247931

[8] Katoh K. and Standley D. M. (2013). MAFFT Multiple Sequence Alignment Software Version 7: Improvements in Performance and Usability. Mol Biol Evol. 30(4): 772 780 780. 10.1093/molbev/mst010 23329690 PMC3603318

[9] Nguyen L. T., Schmidt H. A., von Haeseler A. and Minh B. Q. (2015). IQ-TREE: A Fast and Effective Stochastic Algorithm for Estimating Maximum-Likelihood Phylogenies. Mol Biol Evol. 32(1): 268 274 274. 10.1093/molbev/msu300 25371430 PMC4271533

